# Combined helical tomotherapy and Gamma Knife stereotactic radiosurgery for high-grade recurrent orbital meningioma: a case report

**DOI:** 10.3389/fonc.2023.1273465

**Published:** 2023-10-11

**Authors:** Haomiao Zhang, Hanfeng Wu, Jianjie Lu, Wencheng Shao, Lili Yu

**Affiliations:** ^1^ Department of Radiation Oncology, Harbin Medical University Cancer Hospital, Harbin, China; ^2^ Department of Neurosurgery, Shanghai Gamma Hospital, Shanghai, China; ^3^ Department of Radiation Physics, Harbin Medical University Cancer Hospital, Harbin, China

**Keywords:** orbital meningioma, anaplastic meningioma, surgery, radiation therapy, stereotactic radiosurgery, Gamma Knife

## Abstract

Orbital meningioma is a rare type of orbital tumor with high invasiveness and recurrence rates, making it extremely challenging to treat. Due to the special location of the disease, surgery often cannot completely remove the tumor, requiring postoperative radiation therapy. Here, we report a case of an elderly male patient with right-sided proptosis, visual impairment, and diplopia. Imaging diagnosis revealed a space-occupying lesion in the extraconal space of the right orbit. Pathological and immunohistochemical examination of the resected tumor confirmed it as a grade 3 anaplastic meningioma. Two months after surgery, the patient complained of right eye swelling and a magnetic resonance imaging (MRI) scan showed a recurrence of the tumor. The patient received helical tomotherapy (TOMO) in the postoperative tumor bed and high-risk areas within the orbit with a total dose of 48Gy. However, there was no significant improvement in the patient’s right eye swelling, and the size of the recurrent lesion showed no significant change on imaging. Gamma knife multifractionated stereotactic radiosurgery (MF-SRS) was then given to the recurrent lesion with 50% prescription dose 13.5Gy/3f, once every other day. An imaging diagnosis performed 45 days later showed that the tumor had disappeared completely. The patient’s vision remained unchanged, but diplopia was significantly relieved after MF-SRS. We propose a new hybrid treatment model for recurrent orbital meningioma, where conventional radiation therapy ensures local control of high-risk areas around the postoperative cavity, and MF-SRS maximizes the radiation dose to recurrent lesion areas while protecting surrounding tissues and organs.

## Introduction

Orbital meningiomas account for about 2% of all meningiomas and are divided into primary and secondary tumors (1). Primary orbital meningiomas originate from the arachnoid layer of the optic nerve sheath and represent 30% of all orbital meningiomas (2). Secondary intracranial meningiomas typically originate from the sphenoid ridge and invade the orbit, intracranial space, and intraventricular space, accounting for approximately 70% of orbital meningiomas (3). According to the latest classification scheme of the World Health Organization (WHO), meningiomas can be histologically classified as benign (grade 1), atypical (grade 2), or anaplastic (grade 3) tumors (4). Anaplastic meningiomas are rare, accounting for 1-3% of all meningiomas, and are even rarer when they occur in the orbit (5). Surgery is the preferred treatment for anaplastic meningiomas, but complete resection of anaplastic meningiomas occurring in the orbit is difficult, and the recurrence rate is high. Postoperative adjuvant radiotherapy can improve local control and overall survival in such cases (6–11).

However, we found that postoperative conventional radiotherapy alone is not sufficient to prevent a recurrence, especially for high-grade orbital meningiomas (12, 13). Improving local control and delaying recurrence is a major challenge in cancer treatment. Previous studies have reported that stereotactic radiosurgery (SRS) is effective in treating small residual or recurrent orbital anaplastic meningiomas, especially for those maximum diameter smaller than 3 cm. SRS can achieve a steep dose fall-off at the edge of the target volume, thereby reducing the radiation dose to surrounding organs at risk (OARs) and reducing treatment toxicity (14–16).

Classical Gamma knife treatment for brain malignancies may requires multiple fractionated treatments and repeated headframe fixations, which are invasive and emotionally burdensome for patients. The new generation Leksell Gamma Knife Icon™ system from Elekta Company includes a completely non-invasive mask fixation system with cone beam computed tomography (CBCT) image-guided adaptive precise stereotactic radiosurgery (17, 18). It still maintains a high level of accuracy while improving patient comfort. Another major advantage of the Icon™ system is its top-notch internal motion management and cross-repositioning system, which achieves submillimeter precision in treatment (19, 20). We describe a case of recurrent orbital anaplastic meningioma that received radiotherapy with tomotherapy (TOMO) to the recurrent lesion area and the high-risk area within the orbit for nearly 5 weeks. After that, there was no significant change in the recurrent area, we continued with Gamma Knife treatment using the Icon™ system for the local boost of the recurrent lesion area, which showed significant improvement. This study serves as an implication to effectively treat recurrent orbital meningioma.

## Case report

**Figure 1:**
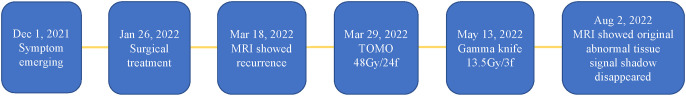
Timeline of onset of patient’s symptoms, comprehensive cancer treatment, and treatment efficacy.

A 71-year-old male patient presented to a hospital other than ours in January 2022, complaining of a one-month history of right eye proptosis, visual impairment, and diplopia (**Figure 1**). Three years prior, the patient underwent surgery to remove a tumor in his right lower limb, which was pathologically confirmed as malignant. The patient received one month of postoperative radiotherapy and has not experienced recurrence since. Twenty years ago, the patient underwent surgery to treat a penetrating injury in his left eye. The patient had no history of hypertension or diabetes and did not smoke or consume alcohol.

Ophthalmic examination revealed a visual acuity of 0.2 in the right eye and 0.3 in the left eye, intraocular pressure of 20mmHg in the right eye and 11mmHg in the left eye, normal eye position and movement, and normal orbital pressure. The right lower eyelid was swollen, and a diffuse mass was palpable below the subcutaneous tissue and the lower margin of the orbit. The mass had a tough texture, unclear boundaries, poor mobility, and no tenderness. Fundoscopy was not possible.

**Figure 2:**
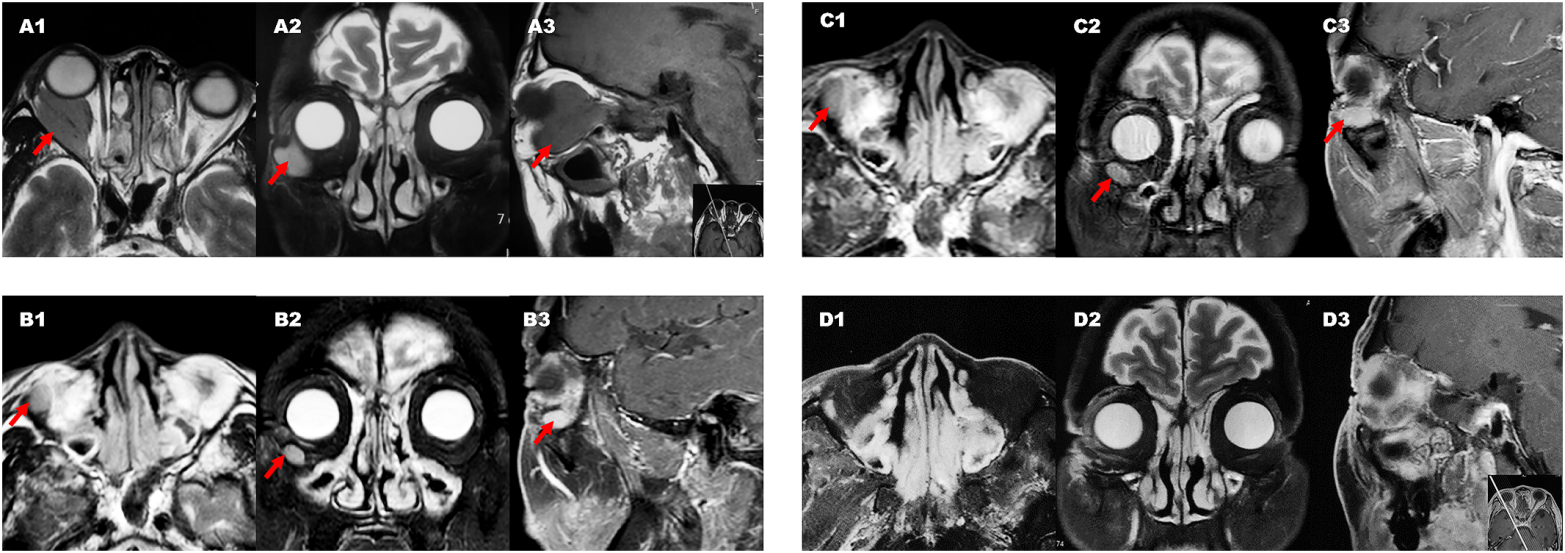
Diagnostic image. Group A (A1, A2, and A3) consists of preoperative MRI images. Group B (B1, B2, and B3) displays MRI images taken before TOMO treatment. Group C (C1, C2, and C3) shows MRI images taken before the Gamma Knife treatment, while group D (D1, D2, and D3) displays MRI images taken after the Gamma Knife treatment. Within each group, 1, 2, and 3 correspond to the transverse, coronal, and sagittal planes, respectively, showing the maximum level of the lesion. The arrow indicates the location of the lesion.

Magnetic resonance imaging (MRI) revealed an irregularly shaped mass measuring 3.5×3.4cm within the intraconal and extraconal spaces of the right eye orbit (**Figures 2A1–3**). The mass had equivalent T1 and T2 signals and restricted diffusion. The mass was found to encompass the lateral rectus muscle, the anterior segment of the intracanalicular optic nerve, and the posterior wall of the eyeball. The tumor abutted the orbital septum anteriorly, causing displacement of the eyeball and anterior migration of the lacrimal gland and the intraorbital fat into the orbital septum. The tumor was removed via an incision through the lateral canthus, and the surgical specimen appeared soft and poorly defined. The tumor had infiltrated the lateral and inferior rectus muscles and was adhered to the optic nerve and other tissues. Immunohistochemical analysis revealed positive expression of vimentin, EMA (2+), AR (3+), CD34, Ki-67 (30-40%), and TNI-1; and negative expression of S100, CK, P53 (few +), desmin, SOX10, STAT6 (plasma cell+), EBER, Syn, CgA, ERG, SMA, β-catenin, HMB45, MelanA, CD31, CD117, Dog-1, WT1, Bc12 (few +), P63, calponin, CK7, CK5/6, CK8/18, PR, and SSTR2.

Postoperative pathological examination confirmed that there was a soft tissue tumor in the right orbital cavity, with cells exhibiting epithelial and short spindle-shaped morphology and approximately 33 mitoses per 10 high-power fields. Combined with the results of immunohistochemical staining, the diagnosis was an anaplastic meningioma, WHO grade 3. Postoperative ophthalmic examination showed that the right eye had a visual acuity index of greater than one meter, normal intraocular pressure and orbital pressure, a normal eye position, ptosis, poor mobility, and slight restriction of eye movement. One month after surgery, MRI showed multiple irregular patchy shadows in the outer lateral part of the right orbital cavity, with small nodules visible inside, which were considered to be postoperative changes. Two months after surgery, a follow-up MRI of the orbital cavity showed a spindle-shaped nodule shadow in the outer inferior region of the right orbital cone, which was isointense on T1-weighted images and slightly hyperintense on T2-weighted images, with uniform enhancement, a long axis of approximately 15mm, smooth edges, and a broad base connected to the adjacent orbital wall (**Figures 2B1–3**). The local abnormal signal in the anterior chamber was slightly increased compared with the previous month. A nodular shadow was seen in the right external rectus muscle area, with a long axis of approximately 9mm and moderate enhancement, but no obvious change. Irregular stripe-like enhancement was observed in the right internal and external rectus muscle areas, with irregular edges. Multiple rich blood supply masses were observed in the right orbital cavity, which was slightly larger than those observed one month earlier, suggesting that partial recurrence after treatment may be possible.

**Figure 3:**
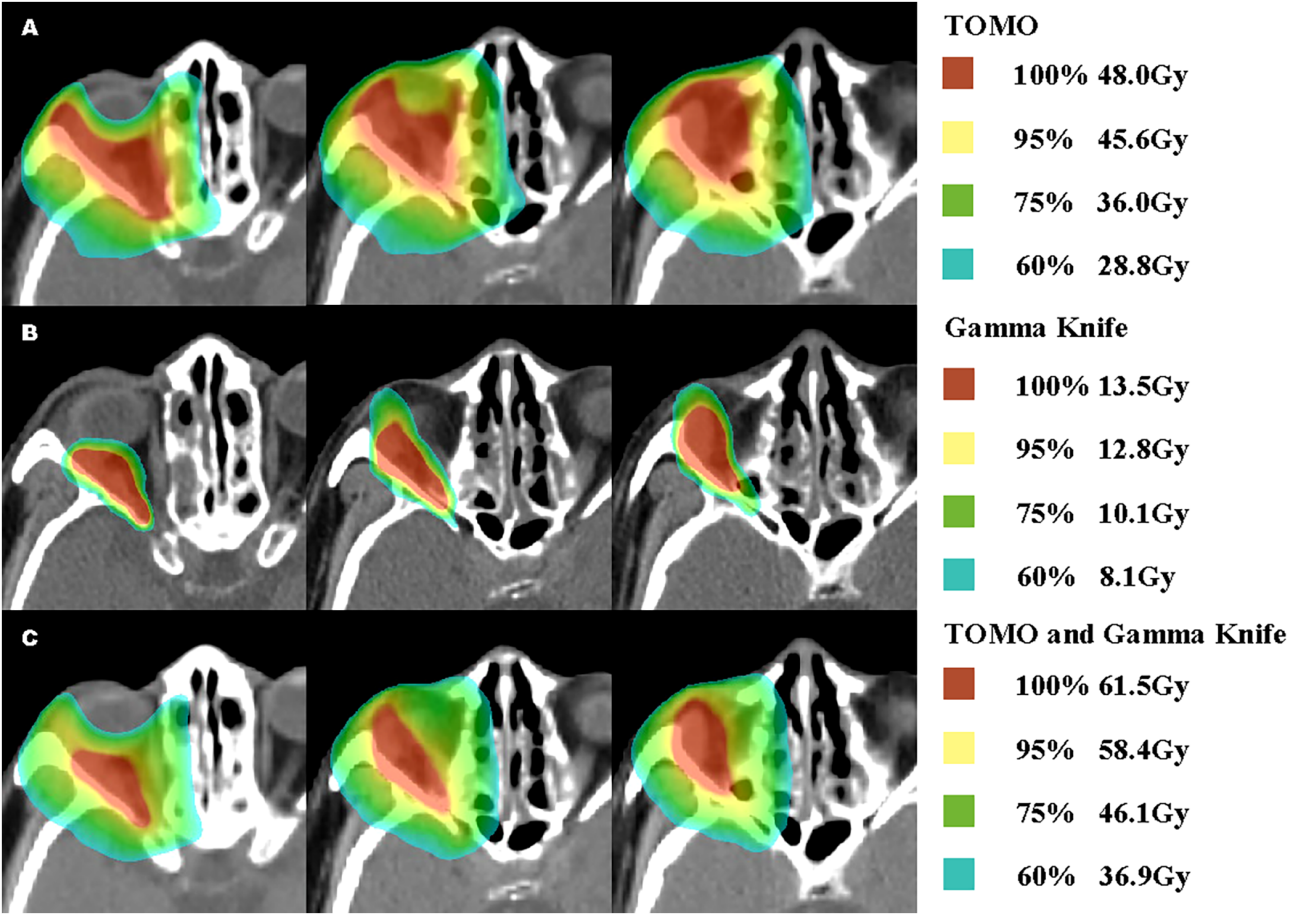
Percentages Dose and Concrete dose Distribution of TOMO, Gamma Knife and Rigid Fusion TOMO and Gamma Knife.

In order to seek further treatment, the patient came to our radiotherapy outpatient clinic in March 2022 and received postoperative and recurrent lesion TOMO radiotherapy in the tumor bed area. CTV60 includes the range of abnormal signals detected with T1-enhanced and fat-suppressed thin cross-sectional MRI images, as well as the range of tumor invasion observed in surgery. CTV54 includes areas of high-risk subclinical lesions. The medial, outer, superior, and lower boundaries of CTV54 are delineated along the orbital wall. The posterior boundary is set to the posterior wall of the orbit and includes the surrounding skull base foramen. Planned target volume (PTV) is generated by a 1mm extension. The prescription dose is 60Gy and the coverage of PTV is 96%. The planning system used is TOMO5.1.16 (with a field width of 2.5cm, pitch of 0.287, and modulation factor of 2.3). The single-fraction prescription dose was 2Gy for the recurrent lesion area and 1.8Gy for the tumor bed area. The treatment process went smoothly, and the patient did not experience any discomfort. After nearly 5 weeks of radiotherapy, a follow-up MRI of the orbital cavity showed no change in the size of the recurrent lesion in the right orbital cavity (**Figures 2C1–3**). The total radiation dose for the recurrent lesion and tumor bed areas was 48Gy and 43.2Gy, respectively. After consultation with colleagues, it was decided to give the patient a combined Gamma Knife treatment for the recurrent lesion and tumor area. The target area of the Gamma Knife treatment is delineated as the range of abnormal signals seen in T1-enhanced and fat-suppressed thin laminar cross-sectional MRI images. The planning system used is Leksell Gamma Knife^®^ 11.1.0. Because of submillimeter target accuracy and a minimum displacement of 1.5 mm, Gamma Knife treatment eliminates the need for margin expansion of the PTV. The 50% prescription dose is 13.5Gy and the coverage of PTV is 91%. On May 13, 2022, Gamma Knife treatment was performed with 50% prescription dose 13.5Gy/3f, once every other day, which was completed within one week (**Figure 3**). Before the Gamma Knife treatment, the patient’s visual acuity was 0.3 in the right eye and 0.2 in the left eye, and there was no change in visual acuity after treatment. MRI from an external hospital on August 2, 2022, showed that the original solid soft tissue signal shadow had disappeared, with the local blurring of the edges of the inner and outer rectus muscles and a few slightly longer T1 signals. The enhancement scan did not reveal any abnormal enhancement. The anterior protrusion of the right eyeball had decreased compared with before. An MRI follow-up on August 2, 2022, showed no significant changes compared with the previous one (**Figures 2D1–3**).

## Discussion

Meningiomas with anaplasia features account for 1-3% of all meningiomas. They are characterized by increased mitotic activity > 20 mitoses per 10 high-power fields (HPF) or obvious developmental abnormalities. Compared with benign meningiomas, they exhibit more invasive behavior, with 6-8 times increased risk of recurrence and a significantly increased risk of death due to tumor progression (5, 21–23). Surgical resection is the preferred treatment, and complete resection is a key factor in local control and survival. However, complete resection is not always possible, especially for meningiomas located in the orbital region, which are difficult to completely remove due to the impact of the surgical approach and surrounding important neurovascular structures. The recurrence rate cannot be ignored, and postoperative adjuvant radiation therapy (PORT) is recommended to increase local control after resection. Since such cases are rare, the recurrence and survival outcomes of anaplastic meningiomas in the orbital region are not yet clear, but studies on anaplasticmeningiomas and low-grade orbital meningiomas provide a reference for us. In studies of anaplasticmeningioma patients who underwent surgical treatment, Jennifer Moliterno and Wei-Dong Tian et al. reached the consensus that the median overall survival (OS) of anaplasticmeningioma patients who underwent surgical treatment was 3.0 years (24, 25). Matthieu Peyre et al. found that the median OS of anaplastic meningiomas.

Meningioma patients was 2.6 years, with a 5-year survival rate of 10% (26). Research on high-grade orbital meningiomas has not yet been conducted, but in studies on low-grade orbital meningiomas, adjuvant radiation therapy was found to be related to local control of the tumor. Nicole Angela Terpolilli et al. divided 122 patients who underwent surgical resection for WHO 1-grade orbital meningioma into a simple surgical treatment group and a postoperative radiation therapy group, with 23 patients receiving postoperative radiation therapy. Patients who received postoperative radiation therapy had significantly later tumor recurrence or progression (76.3 vs 40.7 months) (6). Due to the small number of patients, there are currently no prospective studies to confirm this view. Issues such as the timing of postoperative radiation therapy, optimal radiation therapy techniques, dose, and segmentation methods still need to be further studied.

PORT can be carried out through conventional radiation therapy or SRS. Conventional fractionated radiation therapy is usually used as adjuvant therapy for patients with large resected cavities or large recurrent tumors, while SRS or hypofractionated regimens may represent feasible treatment options for small to moderate tumors, typically those smaller than 3 cm or not in close proximity to sensitive brain structures. There are many critical organs surrounding tumors within the orbit, such as the lens and optic nerve, and to protect this OARs, the dose to certain parts of the tumor may be inadequate, which can result in low local control rates. Given the high risk of local recurrence in areas where the delivered dose is insufficient to meet critical OARs constraints and the potential futility/extreme morbidity of salvage surgery, a safe and effective dose escalation method is needed to increase tumor control. It is difficult to deliver adequate disease control doses using traditional intensity-modulated radiation therapy (IMRT) without causing excessive toxicity. New advanced radiation therapy techniques can provide excellent target dose coverage, precise target localization, and rapid dose delivery.

TOMO is a novel form of IMRT based on computed tomography (CT) imaging. The linear accelerator used in HT can rotate continuously through 360 degrees and has 51 optimized beam angles and a continuously moving treatment couch. Radiation is delivered in the form of helical beams with constant beam widths of 1, 2.5, and 5 cm through spiral transmission scans, equipped with an aerodynamic binary multileaf collimator (MLC) system with fast leaf transition times. HT can shape the radiation dose to conform to the complex shape of the tumor area and avoid delivering high-dose radiation to OARs by rapidly opening and closing the leaves in the collimator rotating around the patient. Currently, helical TOMO makes prescription dose cover more than 98% volume of the tumor and have satisfied conformity. It also has a steep dose fall-off gradient in the surrounding normal tissues, providing better protection for adjacent normal tissues and organs at risk (OARs) (27, 28). Compared to commonly used radiation therapy techniques, such as volumetric-modulated arc therapy (VMAT) and fixed-field IMRT (FF-IMRT), HT achieves the best consistency and uniformity in target coverage, reduces the maximum dose delivered to OARs such as the optic nerve, and has advantages in target coverage and OAR protection (29, 30).

Over the past few decades, SRS has been shown to play an important role in the treatment of meningioma patients. Due to the sharp dose drop-off, normal tissue surrounding the lesion receives significantly less radiation than the target area, limiting the potential toxicity of the treatment. SRS should be considered for meningiomas involving important neurological or vascular structures and for intracranial tumors remaining after surgery. Current stereotactic techniques include Gamma Knife and LINAC-based SRS systems such as CyberKnife. Traditionally, patients treated with LINAC-based SRS systems are fixed in a high-precision, frameless stereotactic mask fixation system, while patients treated with Gamma Knife SRS are placed in a rigid stereotactic frame with submillimeter target accuracy, and Gamma Knife treatment often requires multiple segmentation and fixation of the head frame, which is invasive. The latest developed ICON Gamma Knife technology can use mask fixation, reducing patient pain and injury. Studies have suggested that these two options perform well in terms of geometric accuracy and relative and absolute dosimetry between expected and delivered radiation doses in both framed and frameless treatments (20, 31). Prospective studies have shown that mask fixation does not lead to worse outcomes or an increase in radiation necrosis (32). However, some studies have suggested that mask fixation may result in greater motion variability, which cannot be ignored when treating small lesions or lesions near important structures (33).

In recurrent tumors, SRS can achieve good tumor control, but may result in higher rates of recurrence in the surrounding area. The dose planned for Gamma Knife treatment is not uniform within the prescribed dose, and the actual average dose is higher than the peripheral dose of the tumor. This differs from the IMRT technique and limits the use of Gamma Knife treatment for high-grade orbital meningiomas that invade the optic nerve, as the peripheral dose cannot be increased. However, combining IMRT with Gamma Knife treatment overcomes this limitation. This combined therapy enables higher irradiation doses for the tumor, while maintaining safe doses for the organs at risk (OARs), thus enhancing the possibility of tumor control. We rigidly fused TOMO with Gamma Knife to obtain the corresponding cumulative dose (**Table 1**). Although the algorithms for TOMO and GK are actually different, the cumulative dose shown by rigid fusion may not be entirely accurate enough, but it can still be used as a reference to some extent. In our report, we adopted a new pattern of IMRT combined with MF-SRS for recurrent tumors. The dose of the new IMRT ensured control of the entire postoperative area, while the MF-SRS ensured high-level conformity for recurrent tumors in the orbit, providing a sufficient dose for the recurrent tumor while minimizing toxicity and protecting important structures such as the optic nerve and lens. We report a case of recurrent malignant brain meningioma located in a complex anatomical position under the orbit treated with the new IMRT combined with MF-SRS.

**Table 1:** The Dose Distribution of OARs for Each Treatment.

Name	TOMO		Gamma Knife		Rigid Fusion of TOMO and Gamma Knife	
	Max Dose (Gy)	Mean Dose (Gy)	Max Dose (Gy)	Mean Dose (Gy)	Max Dose (Gy)	Mean Dose (Gy)
Eye-R	51.26	30.62	19.60	4.80±2.10	66.30	34.38
Len-R	10.62	5.12	3.50	2.90±0.20	18.40	8.37
OpticNerve-R	51.88	50.28	11.20	5.00±2.30	67.34	56.65
OpticChiasm	43.10	21.38	1.20	0.60±0.30	45.38	22.60
Pituitary	22.47	17.34	0.70	0.50±0.10	24.62	18.50

OARs, Organs at Risk; TOMO, Tomotherapy.

The optimal dose for PORT in meningiomas remains unclear. Higher RT doses appear to improve local tumor control in patients with anaplasia meningiomas. Some studies suggest using higher margin doses (> 13 Gy) to achieve better local control (34). For patients receiving SRS, a single dose of 14-18 Gy is typically used at most radiation centers for similar local control, while doses ≤ 12 Gy are associated with poorer local control rates (35), and doses ≥ 10 Gy to the optic apparatus are associated with decreased vision after SRS (36). Low fractionated treatment for meningiomas around the optic nerve can deliver SRS doses of 19.5 Gy/3 fractions and 25 Gy/5 fractions to the optic pathway while still maintaining a lower risk of optic neuropathy and the typical range for radiation therapy margin doses in WHO grade 3 meningiomas are 18 to 24 Gy (37). Additionally, a recent study on late-stage head and neck cancer patients proposed a novel treatment approach of IMRT plus SRS for local boost, advocating for a dose escalation of 50-54 Gy (fractionation of 1.6-2.0 Gy per fraction) for patients with R1 positive margins adjacent to critical structures under microscopic examination, with a SRS boost dose of 8-10 Gy to achieve an EQD2 (radiation equivalent dose) (α/β=10) cumulative total dose of at least 60-66 Gy, limited by critical structure tolerance (38).

In this case report, the patient’s follow-up time is relatively short, and continued follow-up is necessary to clarify the long-term efficacy of this new treatment regimen. Currently, the patient has not experienced recurrence, and their vision has not been affected. Due to the rarity of this case, the treatment experience and effectiveness for this patient are highly valuable, and we hope that this treatment regimen can be better applied to the treatment of high-grade malignant tumors in these rare and special locations.

## Conclusions

Treating postoperative recurrent refractory malignant meningioma in the orbit with IMRT plus MF-SRS local boost therapy may be a new safe and effective treatment modality that can achieve good local control and preserve visual function.

## Patient’s perspective

The patient provided fully informed consent for the publication of this report and the accompanying images. The patient is highly satisfied with the oncological, functional, and cosmetic outcomes obtained.

## Data availability statement

The original contributions presented in the study are included in the article/supplementary material. Further inquiries can be directed to the corresponding authors.

## Ethics statement

Ethical review and approval were not required for this study involving a human participant. The patient provided his written informed consent to participate in this study. Written informed consent was obtained from the individual for the publication of any potentially identifiable images or data included in this article.

## Author contributions

HZ: Data curation, Visualization, Writing – original draft. HW: Supervision, Validation, Visualization, Conceptualization, Writing – review & editing, Formal Analysis, Methodology, Software. JL: Formal Analysis, Visualization, Methodology, Writing – review & editing. WS: Conceptualization, Writing – review & editing. LY: Methodology, Writing – review & editing.
